# Effects of WhatsApp reminder-supported mental contrasting with implementation intentions on university students’ self-efficacy in sport training

**DOI:** 10.1038/s41598-026-46181-x

**Published:** 2026-03-27

**Authors:** Yalin Aygun, Huseyin Gurer, Sakir Tufekci, Cemil Colak, Emek Guldogan, Burak Yagin, Hasan Ucuzal, Burak Canpolat, Nouf H. Alkhamees, Sameer Badri AL-Mhanna

**Affiliations:** 1 Department of Sport Management, Faculty of Sport Sciences, Inonu University, Malatya, 44280 Turkey; 2 Department of Coaching Education, Faculty of Sport Sciences, Inonu University, Malatya, 44280 Turkey; 3 Department of Biostatistics, Faculty of Medicine, Inonu University, Malatya, 44280 Turkey; 4 Department of Physical Education and Sport on Disabilities, Faculty of Sport Sciences, Inonu University, Malatya, 44280 Turkey; 5 Department of Rehabilitation Sciences, College of Health and Rehabilitation Sciences, Princess Nourah bint Abdulrahman University, P.O. Box 84428, Riyadh, 11671 Saudi Arabia; 6 Department of Physiology, Saveetha Medical College and Hospital, Saveetha Institute of Medical and Technical Sciences, Chennai, Tamil Nadu India; 7 Department of Physiology, School of Medical Sciences, University Sains Malaysia, Kubang Kerian, 16150 Kelantan Malaysia; 8 Department of Higher Studies, Al-Qasim Green University, Babylon, Iraq

**Keywords:** Self-regulation, Self-efficacy, Mental contrasting, Implementation intentions, Digital reminders, Sport training, Health care, Psychology, Psychology

## Abstract

Mental contrasting with implementation intentions (MCII) is an effective self-regulation strategy for goal pursuit. Despite growing interest in MCII within behaviour change research, relatively little is known about how such self-regulation strategies operate in real-world sport training environments or whether simple digital reminders may support their enactment. This exploratory randomized intervention study examined whether adding WhatsApp reminder messages to a contextually adapted MCII intervention was associated with changes in university students’ self-efficacy in tennis training. A total of 31 university students from a university tennis club were randomly assigned to either an MCII-only condition or an MCII intervention combined with WhatsApp reminder messages delivered prior to training sessions. Self-efficacy was assessed before and after a four-week intervention period using the General Self-Efficacy Scale (GSES). Within-group analyses did not reveal statistically significant changes in self-efficacy in either intervention condition. However, between-group comparison of change scores indicated a significant difference favouring the reminder-supported MCII condition (*U* = 94.50, *p* = 0.015), although the estimated effect size should be interpreted cautiously given the small sample. Session attendance did not differ between groups, suggesting that differences in intervention exposure were unlikely to account for the observed outcome. These findings suggest that integrating simple digital reminder messages into MCII-based interventions may support the application of self-regulation strategies in sport training contexts. Given the exploratory design and limited statistical power, the findings should be interpreted cautiously and require confirmation in larger, prospectively registered trials.

**Supplementary Information:**

The online version contains supplementary material available at 10.1038/s41598-026-46181-x.

## Introduction

Whether performed occasionally or in an organized form, participation in sport is linked to the promotion of public health and improvements in physical and mental fitness^1,2^. Early participation in regular and optimally pursued sporting activities increases the likelihood of maintaining high levels of physical functioning throughout the lifespan^3,4^. Indeed, healthy lifestyles followed in one’s early 20s tend to be associated with positive health behaviours later in life^5^. At this stage, understanding the effect of factors on current sport participation levels, as well as future participation and adherence, is of critical importance.

Based on the findings of a recent systematic review by Kovács and Szakál^6^, factors influencing sport participation show considerable variety and are detected at the individual (e.g., gender, age, positive psychological factors), micro- (e.g., family, peers, and coaches), meso- (e.g., climate), or macro level (e.g., cultural, societal, and institutional influences). The effects of many individual-level psychological factors (e.g., self-efficacy), particularly in interaction with self-regulation processes, has received relatively greater research attention from academics^7,8^. Self-regulation is explicitly posited by Bandura^9^ as a key determinant of success in sport participation. Whilst self-efficacy, social support, and outcome expectations are considered important for maintaining a physically active lifestyle, Bandura^10^ proposed self-regulatory behaviour as an essential feature of living organisms that allows them to adapt changing circumstances.

Self-regulation can be defined as a goal-directed and adaptive process that enables individuals to plan, monitor, and adjust their emotions, thoughts, and behaviours in pursuit of desired outcomes^11,12^. Thus, self-regulation is associated with self-knowledge, the ability to evaluate and improve one’s own skills, and the purposeful regulation of one’s own activity. From a social cognitive perspective, self-regulation is not viewed as a fixed personality trait but rather as a dynamic and cyclical process unfolding through three interrelated phases^13^: forethought (task analysis, setting goals, selecting a strategy), performance or volitional control (self-control, self-instruction, visualization, creating images), and self-reflection (self-evaluation, self-judgment, self-determination). Self-regulation skills can be enhanced through deliberate behavioural management, including planning activities, monitoring their implementation, and adjusting behaviour when necessary^14^.

As self-regulation supports the organization of activities, the regulation or inhibition of behaviour, and effective responses to challenging conditions, it holds clear relevance for sport practice^15–17^. Scholars are beginning to explore the links between self-regulation and deliberate practice in the search for an answer to the inquiry of what ensures development and expertise^17^. Although the phenomenon of self-regulation is not new and several researcher-developed self-regulatory strategies have been proposed, imagery strategies such as mental contrasting and implementation intentions (MCII) often involve limited extrinsic rewards in sport research practice^18^.

### MCII as a self-regulation strategy in sport training

Mental contrasting is a self-regulatory imagery strategy in which individuals envision a desired future and contrast it with present constraints, thereby highlighting the discrepancy that prompts goal-directed action^19^. Mental contrasting unfolds in three steps that initiate goal-directed action: (1) defining an important wish; (2) identifying and vividly imagining the best possible outcome of its fulfilment; and (3) identifying and mentally elaborating an obstacle in the present reality that stands in the way of attaining the desired future^20,21^.

Mental contrasting derives from fantasy realization theory^20,21^, which posits it as a self-regulatory imagery strategy for initiating behaviour change. Mental contrasting, which juxtaposes thoughts of a desired future with present reality, is hypothesized to energize goal pursuit when success is perceived as feasible and to disengage effort when the desired future is perceived as unattainable^20,22^. A series of experimental findings suggests that mental contrasting operates through nonconscious cognitive mechanisms that frame present reality as an obstacle, heighten awareness of relevant barriers, and strengthen the association between desired future states and current conditions^23–25^. Mental contrasting’s effectiveness has been documented using self-report, observational, and experimental methods across a range of outcomes, including planning, anticipated disappointment following failure, and action initiation^20,21,26–28^.

Implementation intentions^29^, formulated as “if (obstacle)–then” plans, strengthen the associative link between obstacles and instrumental behaviour^30^. They operate by prompting individuals to specify a situational cue (e.g., “if I feel too tired before tennis practice”) and link it to an appropriate goal-directed response (e.g., “then I will still attend training and complete the warm-up”). With the emergence of the specified cue, implementation intentions activate automatic action control, facilitating goal attainment^31,32^. Studies have shown that implementation intention effects increase when plans adopt a contingent “if–then” format, participants are highly motivated, and plans are rehearsed^33^. In the context of physical activity, a meta-analysis by Belanger-Gravel et al.^34^ identified stronger effects of implementation intentions among students and in interventions incorporating barrier management. These findings highlight the relevance of examining implementation intention mechanisms within university student populations and structured sport training contexts such as tennis.

When combined with implementation intentions, mental contrasting may strengthen goal-directed planning by identifying the obstacle that defines the “if” component and the instrumental response that specifies the “then” component of the plan^23,30^. Thus, MCII integrates two theoretically complementary strategies. Kirk et al.’s^35^ study found that the MCII strategy led dyads to reach the largest joint agreements compared to dyads that used only mental contrasting or implementation intentions.

Sport training contexts such as tennis may offer a theoretically meaningful pathway for examining the effects of MCII. The structure of tennis practice naturally engages multiple sources of self-efficacy, including mastery experiences derived from skill improvement, observational learning through modelling and coaching demonstrations, social persuasion via instructor feedback, and physiological or affective responses associated with training and competition^9,36,37^. In this regard, MCII represents an integrated self-regulation strategy that combines mental contrasting with implementation intentions to facilitate goal-directed behaviour. Digital reminder messages delivered via WhatsApp may further strengthen the effects of MCII by supporting the enactment of implementation intentions in sport practice^38,39^.

### Self-efficacy in sport participation

Self-efficacy is a construct that focuses on “people’s judgements of their capabilities to organize and execute courses of action required to attain designated types of performances”^40^(391). A crucial aspect of self-efficacy is that efficacy beliefs shape individuals’ choice of activities, the effort they invest, and their persistence when confronted with challenges^36^. These beliefs reflect individuals’ perceived capability to perform a specific task rather than their actual abilities or performance^41^.

To explain the origins of self-efficacy beliefs, Bandura^9,36^ identified four primary sources: mastery experiences, vicarious experiences, verbal persuasion, and physiological and affective states. Mastery experiences emerge from individuals’ reflections on their own prior task accomplishments, whereas vicarious experiences develop through observing the performance of others. Verbal persuasion involves evaluative judgments or encouragement communicated by significant others, while physiological and affective states relate to the interpretation of bodily reactions and emotional responses during task engagement. These sources collectively explain how efficacy beliefs emerge, evolve, and become strengthened through experience.

In this study, the MCII intervention does not directly manipulate the four sources of self-efficacy. Instead, it operates indirectly by strengthening these sources within the context of tennis training. The intervention primarily facilitates mastery experiences by promoting regular participation in practice, thereby increasing opportunities for successful task execution. WhatsApp reminder messages may additionally provide elements of verbal persuasion through motivational prompts and encouragement. Effects on physiological or affective states may also emerge indirectly as participants develop greater confidence during practice, whereas vicarious experiences are not explicitly targeted because the intervention does not incorporate observational learning or modelling components.

Self-efficacy supports sport participation by shaping self-regulatory processes involved in goal setting and behavioural regulation^37,42^. In addition to self-efficacy, other motivational processes^43,44^(e.g., autonomous motivation, goal commitment, outcome expectancies, and perceived social support) and individual factors (e.g., goal motives^45,46^, threat appraisals^46^ may also influence the effectiveness of self-regulation strategies in sport contexts. Autonomous motivation, defined in self-determination theory as engagement driven by intrinsically valued or self-endorsed reasons, supports volitional behavioural regulation^47,48^. Under such conditions, individuals are more likely to enact their intentions in sustained goal-directed engagement during sporting activities^49^.

### Study purpose and hypotheses

The purpose of this study was to examine whether a contextually adapted MCII intervention, implemented either alone or in combination with WhatsApp reminder messages, is associated with changes in university students’ general self-efficacy within the context of tennis training. In addition, the study explored whether the MCII intervention combined with WhatsApp reminder messages results in more favourable changes in self-efficacy than the MCII intervention implemented alone. Given the exploratory nature of the study, the following hypotheses were formulated to guide the investigation rather than to provide confirmatory tests.

H1. The MCII intervention combined with WhatsApp reminder messages is expected to produce more favourable changes in university students’ general self-efficacy during tennis training than the MCII intervention implemented alone.

H2. The MCII intervention implemented alone may be associated with changes in university students’ general self-efficacy during tennis training.

H3. The MCII intervention combined with WhatsApp reminder messages may be associated with changes in university students’ general self-efficacy during tennis training.

## Methods

### Study design

This prospective experimental study explored the preliminary effectiveness of a contextually adapted MCII intervention in enhancing university students’ general self-efficacy within the context of tennis training. In addition, it examined whether augmenting MCII with structured WhatsApp reminder messages produced greater improvements in self-efficacy compared with the MCII procedure implemented alone. A two-arm randomized intervention design with pretest–post-test assessments was implemented, with participants allocated in a 1:1 ratio to either (a) MCII-only or (b) MCII combined with reminder messages (MCII + Reminders).

Given the small sample size, convenience sampling frame, and absence of an a priori power calculation, this study should be considered exploratory and pilot in nature. Accordingly, the findings should be interpreted as hypothesis-generating rather than confirmatory, and replication in larger, pre-registered trials will be required before definitive conclusions can be drawn. Consistent with the exploratory nature of the study, the hypotheses were formulated to guide the investigation rather than to provide confirmatory tests.

This trial was retrospectively registered at ClinicalTrials.gov (Identifier: NCT07427030) on 20 February 2026, after data collection had been completed, and the study is reported in accordance with CONSORT guidelines^50^. Registration was completed retrospectively because the study was initially designed as an exploratory pilot intervention rather than a confirmatory clinical trial. As the study was retrospectively registered, prospective protocol verification was not possible, which represents a methodological limitation. Future studies should ensure prospective trial registration prior to participant enrolment.

The study was conducted in accordance with the Declaration of Helsinki and received ethical approval from the Ethics Committee for Human Research in Educational Sciences at Erzincan Binali Yildirim University (Protocol No. 02/14; Official Document No. E-88012460-050.01.04-153963). Written informed consent was obtained from all participants prior to data collection. Participation was voluntary, and participant confidentiality was maintained throughout the trial. Potential harms and unintended effects were monitored through participant self-report during training sessions. No adverse events or unintended effects related to the intervention were reported.

### Participants and setting

Eligibility criteria required participants to be registered members of the university tennis club, actively participating in the structured training program, to provide informed consent, and to complete baseline assessments. Participants were recruited from a public university tennis club at Inonu University, Malatya, Türkiye. From an initial pool of 42 eligible club members, 31 provided informed consent and were enrolled in the study.

During the intervention period, nine participants withdrew or attended irregularly and were therefore excluded from the final analysis. The final analytical sample comprised *n* = 22 participants who completed all study phases, including baseline assessment, intervention exposure, and post-test evaluation. The sample included 17 women and 5 men aged 18–31 years. Detailed demographic characteristics are presented in Table 1.

**Table 1:** Baseline characteristics of participants.

Variable	*n*	%
Gender		
Female	17	77.3
Male	5	22.7
Age		
Range	18–31	
Undergraduate year level		
Year 1	7	31.8
Year 2	7	31.8
Year 3	7	31.8
Year 4	1	4.5

**Fig. 1:**
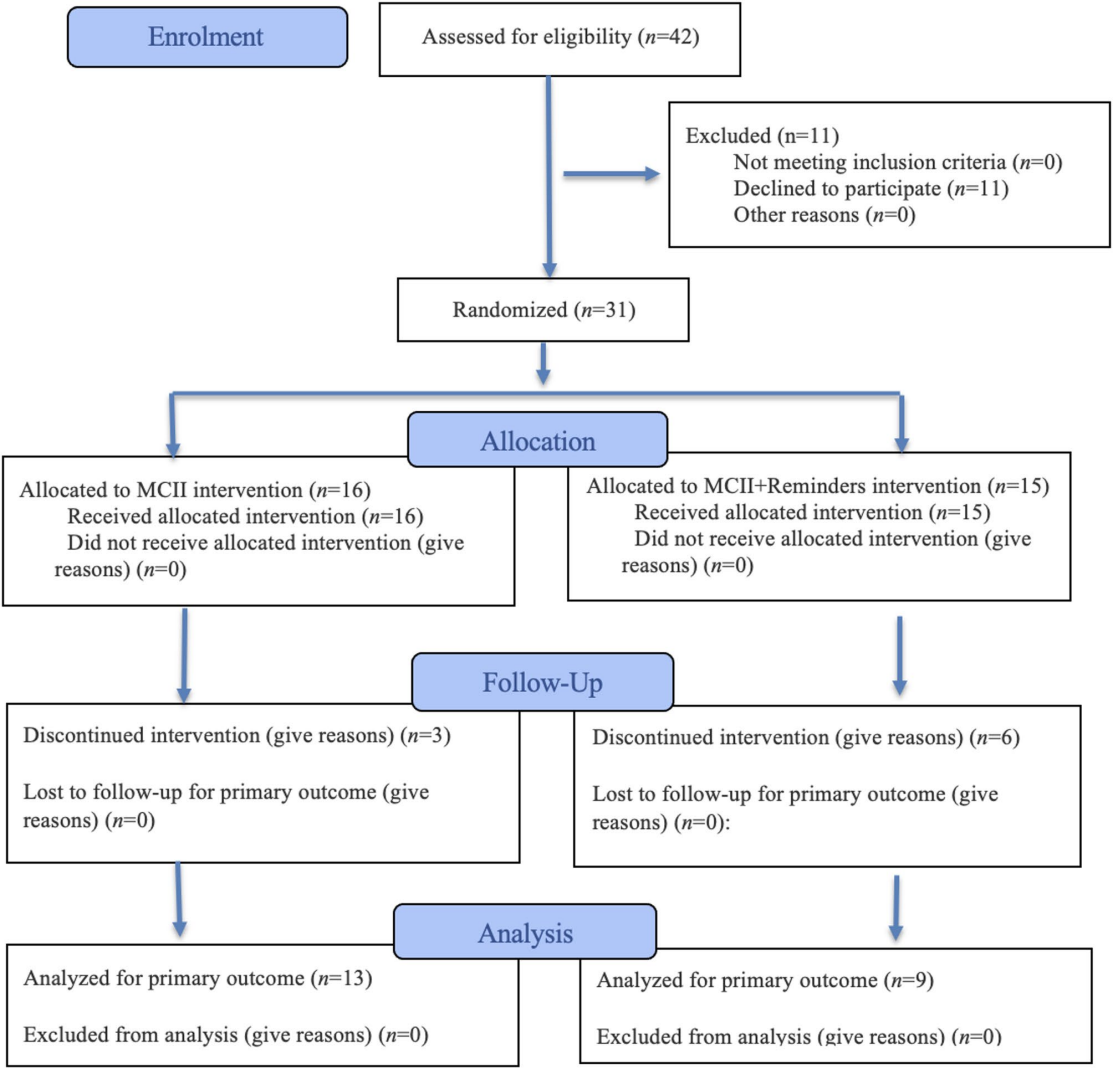
CONSORT 2025 flow diagram^50^.

Of the 31 enrolled participants, *n* = 9 were excluded prior to analysis, including *n* = 5 from the MCII-only condition and *n* = 4 from the MCII + Reminders condition. Reasons for exclusion included irregular attendance (failure to attend at least one training session) and failure to complete post-test measures. The final analytical samples were *n* = 13 (MCII-only) and *n* = 9 (MCII + Reminders). All participants allocated to the intervention conditions received the assigned intervention prior to attrition. Condition-specific attrition is illustrated in the CONSORT flow diagram (Fig. 1).

Among the nine excluded participants, available baseline self-efficacy data (collected for *n* = 7 prior to withdrawal) indicated a median GSES score of 3.30 (*IQR* = 3.10–3.50), compared with 3.40 (*IQR* = 3.30–3.90) among completers. Given the small number of non-completers with available baseline data, formal statistical comparison was not feasible. However, descriptive inspection of available baseline characteristics, including age, gender distribution, and baseline self-efficacy scores, did not suggest pronounced systematic differences between completers and non-completers, although potential attrition bias cannot be excluded.

#### Sample size and randomization

Recruitment was restricted to a naturally occurring university tennis club cohort; therefore, an a priori sample size calculation was not feasible. A convenience sampling strategy was adopted based on the available participant pool. Participants were assigned to study conditions using a computer-generated simple randomization procedure implemented in SPSS, without blocking or stratification. The allocation sequence was generated by an independent researcher (C.G. and E.G.) who was not involved in participant enrolment or outcome assessment. Allocation concealment was maintained through sequentially numbered, sealed opaque envelopes. These envelopes were opened by the enrolment coordinator only after completion of baseline assessments, and personnel responsible for participant recruitment had no prior access to the allocation sequence. Blinding of participants and instructors was not feasible due to the behavioural nature of the intervention. Outcome assessors were also aware of group allocation, which represents a potential source of detection bias. Analyses were conducted on a per-protocol basis, including only participants who completed all intervention and assessment phases.

With the final sample sizes, the study had statistical sensitivity only for detecting large effects (approximately *d* ≥ 1.10). Effects in the small-to-moderate range (*d* = 0.20–0.80), which are common in psychological intervention research, would not have been detectable with the available sample. Null findings should therefore be interpreted cautiously, as the study was underpowered to exclude smaller yet potentially meaningful effects. A post-hoc sensitivity analysis was conducted to contextualize this limitation rather than to demonstrate statistical adequacy.

### Intervention

The intervention was designed as a micro-dose self-regulation program consisting of brief, repeated cognitive exercises integrated into regular tennis training sessions rather than delivered as a high-intensity standalone intervention. It was implemented over four weeks alongside the routine training schedule (two sessions per week). Prior to the intervention phase, all participants received standardized instruction on the MCII strategy. Both study conditions completed the same core cognitive activity; the only difference between groups was the presence or absence of digital reminder messages.

The MCII framework combines mental contrasting, which involves identifying desired outcomes and potential obstacles, with implementation intentions that require forming concrete “if–then” action plans to support goal-directed behaviour. Intervention materials were adapted to the tennis training context by experts in instructional technology and sport sciences. Because attendance was voluntary, participants completed between one and three MCII exercises depending on training participation. Intervention exposure was recorded as the number of completed MCII sessions and considered during data interpretation. The intervention worksheet was administered at the beginning of each attended training session. Intervention fidelity was monitored through attendance records and verification of worksheet completion. No additional interventions beyond routine tennis training were introduced during the study period. In the MCII-only condition, participants completed a mean of 2.8 sessions (*SD* = 0.6; range 2–3). In the MCII + Reminders condition, participants completed a mean of 3.1 sessions (*SD* = 0.6; range 2–4). These values reflect sessions in which the worksheet was completed and verified.

#### MCII

Participants assigned to the MCII condition completed a structured paper-based worksheet immediately before each tennis training session. The worksheet operationalized the MCII strategy and guided participants through two sequential cognitive steps tailored to the training context. First, participants performed mental contrasting by identifying a specific goal for the upcoming session and reflecting on potential internal or external obstacles that could interfere with performance. This step aimed to strengthen goal commitment by linking desired outcomes with realistic constraints. Second, participants formulated implementation intentions by generating concrete “if–then” action plans specifying how they would respond to anticipated obstacles during practice. Participants were instructed to identify a specific cue (e.g., fatigue, loss of concentration, or technical difficulty during drills) and to formulate a corresponding behavioural response (e.g., adjusting practice focus, repeating a drill, or increasing effort). Worksheet structure, instructions, and response prompts were standardized across sessions to ensure consistent intervention delivery. Worksheets were completed at the beginning of each attended session and collected to verify completion. The intervention was embedded within the routine tennis training schedule and did not replace or modify regular practice activities. The structured worksheet used to guide the MCII procedure during training sessions is provided in the Supplementary Material (Supplementary File S1).

#### MCII + reminders

Participants assigned to the MCII + Reminders condition completed the same MCII worksheet protocol as the MCII-only group. The only procedural difference was the addition of reminder messages delivered via WhatsApp. Reminder messages were sent one day before each scheduled tennis training session to prompt anticipation of the upcoming session and encourage preparation for the MCII exercise. These messages were intended to reinforce anticipatory self-regulation without introducing additional cognitive components. The MCII worksheet procedure was identical across both conditions; thus, the presence of digital reminders represented the sole experimental manipulation. Participants in the MCII + Reminders condition received one standardized reminder message via WhatsApp prior to each scheduled tennis training session. Messages were delivered approximately 24 h before the session and were identical in structure across the intervention period. The reminders briefly prompted participants to recall their previously formulated MCII plan and to mentally prepare for the upcoming training session. A typical reminder message read: “Tomorrow’s tennis training is approaching. Please take a moment to recall your goal, the obstacle you identified, and the ‘if–then’ plan you prepared to address it.” Messages were informational and motivational in tone and did not introduce additional behavioural instructions beyond the MCII worksheet procedure.

### Study procedure

Participants were informed about the study procedures and provided informed consent prior to the first training session. Following enrolment, baseline assessments were completed, including demographic information and pretest measures of self-efficacy and related variables. Participants were then randomly assigned to either the MCII-only or the MCII + Reminders condition. Both groups completed a four-week intervention integrated into the routine tennis training schedule. Post-test assessments were conducted immediately after the intervention period. Participant flow throughout the trial is presented in Fig. 1.

### Outcome measures

The prespecified primary outcome was university students’ general self-efficacy within tennis training context. Self-efficacy was assessed at two time points: baseline (pretest) and immediately after completion of the intervention period (post-test). No prespecified secondary outcomes were defined.

Self-efficacy was measured using the General Self-Efficacy Scale (GSES) developed by Schwarzer and Jerusalem^51^. A validated Turkish adaptation of the scale by Aypay^52^ was used in the present study. The instrument consists of ten items rated on a 4-point Likert scale and assesses individuals’ perceived capability to cope with challenges and perform goal-directed behaviours. Previous research with Turkish adult samples has demonstrated acceptable psychometric properties of the scale, including internal consistency coefficients ranging from *α* = 0.80 to 0.83 and evidence of construct validity. In the present sample, internal consistency of the GSES was Cronbach’s *α* = 0.81 at pretest and *α* = 0.83 at post-test, indicating good reliability. The GSES was used because the MCII intervention targets general self-regulatory processes (e.g., goal setting, obstacle anticipation, and action planning) rather than sport-specific performance skills^23,30^.

In addition to the primary outcome, baseline assessments included demographic variables (age, gender, and year of study), as well as training-related expectations and perceived importance of participation. These variables were measured using single-item 7-point Likert-type questions adapted from previously published protocols in physical activity research. Specifically, comparable measures of training expectations and perceived value have been used in studies by Sur^53^ and Liau et al.^54^. These variables were used to characterize the sample and to examine baseline equivalence between intervention conditions.

### Data management and statistical analysis

All study data were collected using standardized paper-based forms and subsequently entered a digital dataset for analysis. Each participant was assigned an anonymized identification code to ensure confidentiality. Data entry accuracy was verified through double-checking procedures. Only participants who completed both baseline and post-test assessments were included in the primary analytical dataset. The final dataset was securely stored and accessible only to the research team.

All statistical analyses were performed using IBM SPSS Statistics, Version 28.0 (IBM Corp., Armonk, NY, USA; https://www.ibm.com/products/spss-statistics). Although preliminary screening did not indicate severe violations of normality, nonparametric methods were selected due to the limited sample size and the ordinal nature of several variables. Analyses proceeded in three sequential steps. First, baseline equivalence between the MCII and MCII + Reminders groups was evaluated using the Mann–Whitney *U* test on pre-intervention measures, including self-efficacy and training-related variables. Second, within-group changes in self-efficacy from pretest to post-test were examined separately for each condition using the Wilcoxon signed-rank test. Third, between-group differences in change magnitude were assessed using the Mann–Whitney *U* test applied to change scores (post-test – pretest).

Effect sizes were calculated for all primary comparisons. The rank-biserial correlation (*r*) obtained from nonparametric tests was converted to Cohen’s *d* using the formula *d* = 2*r* / √(1 − *r*²), following Rosenthal and Rubin^55^, to facilitate interpretation using standardized mean difference metrics. The resulting effect size (*d* = 1.22) should be interpreted cautiously given the small sample size. Change scores were used for between-group comparisons because baseline equivalence was confirmed and the limited sample precluded stable estimation of mixed-effects or ANCOVA models. Statistical significance was set at *p* < 0.05 and all tests were two-tailed. Analyses were conducted using complete-case data, and missing values were not imputed due to the small sample size.

An exploratory intention-to-treat (ITT) sensitivity analysis was additionally conducted using a last-observation-carried-forward approach in which excluded participants were assigned their baseline values. The ITT Mann–Whitney *U* comparison of change scores yielded *U* = 147.50, *p* = 0.076, *r* = 0.32, *d* = 0.67. The attenuated and non-significant ITT result reflects the conservative nature of the imputation procedure, and the per-protocol analysis therefore remains the primary analysis.

## Results

### Baseline characteristics and group comparability

Baseline comparability between the two intervention conditions was assessed using the Mann–Whitney *U* test (Table 2). No statistically significant differences were observed between the MCII and MCII + Reminders groups in self-efficacy (*U* = 36.50, *p* = 0.14), expectation (*U* = 47.50, *p* = 0.44), or perceived importance (*U* = 58.50, *p* = 1.00). Median values and interquartile ranges were similar across groups. Effect sizes for baseline differences were *r* = 0.31 (*d* = 0.66) for self-efficacy, *r* = 0.17 (*d* = 0.34) for expectation, and *r* = 0.00 (*d* = 0.00) for perceived importance. Given the small sample size, the absence of statistically significant differences should not be interpreted as evidence of true equivalence between groups.

**Table 2:** Baseline comparisons between the MCII and MCII + reminders groups.

Variable	MCII mdn (IQR) (*n*_1_ = 13)	MCII + Reminders mdn (IQR) (*n*_2_ = 9)	U	*p*
Self-efficacy	3.4 (3.3–3.9)	3.4 (3.3–3.8)	36.50	0.14
Expectation	6.0 (5.0–7.0)	5.0 (5.0–7.0)	47.50	0.44
Importance	6.0 (5.0–6.5)	6.0 (5.0–7.0)	58.50	1.00

Demographic characteristics were also comparable between conditions. Gender distribution was similar (MCII-only: 10 females and 3 males; MCII + Reminders: 7 females and 2 males; Fisher’s exact *p* = 1.00), and year-level distribution did not differ significantly between groups (*p* = 0.89). These findings indicate no detectable demographic imbalance at baseline, although simple randomization without stratification cannot guarantee balance in small samples.

### Within-group changes in self-efficacy

Changes in self-efficacy from pretest to post-test were examined within each intervention condition using the Wilcoxon signed-rank test (Table 3). No statistically significant within-group changes were observed in either condition. In the MCII-only group, median scores decreased slightly from 3.40 at pretest to 3.20 at post-test (*Z* = − 0.44, *p* = 0.66). In the MCII + Reminders group, median scores remained stable at 3.40 across both measurement points (*Z* = − 0.24, *p* = 0.81).

**Table 3:** Within-group pretest–post-test comparisons of self-efficacy scores.

Group	Pretest mdn	Post-test mdn	Z	*p*
MCII (*n*_*1*_ = 13)	3.40	3.20	− 0.44	0.66
MCII + Reminders (*n*_*2*_ = 9)	3.40	3.40	− 0.24	0.81

These results should be interpreted with caution given the limited statistical power of the study. The sample was powered to detect only large effects (*d* ≥ 1.10). Consequently, smaller but potentially meaningful changes in self-efficacy may not have been detectable in the present sample. The absence of statistically significant within-group effects should therefore not be interpreted as evidence that the interventions produced no change. Accordingly, the between-group comparison represents the primary analytical focus of the study.

### Between-group differences in change scores

The primary test of the intervention’s comparative efficacy examined differences in pretest–post-test change scores between groups using the Mann–Whitney *U* test (Table 4). Participants in the MCII + Reminders condition showed a positive median change score in self-efficacy of 0.20 points (IQR = 0.00–0.20), whereas the MCII-only group exhibited a median decrease of 0.30 points (IQR = − 0.45–0.00). The between-group difference in change scores was statistically significant (*U* = 94.50, *p* = 0.015) and associated with a large effect size (*r* = 0.52, *d* = 1.22). In absolute terms, the MCII-only group showed a median decrease of 0.30 GSES points (approximately 7.5% of the 1–4 scale range), whereas the MCII + Reminders group showed a median increase of 0.20 points (approximately 5.0% of the scale range). The absolute between-group difference was therefore approximately 0.50 GSES points (12.5% of the scale range), although the clinical significance of this difference remains uncertain.

**Table 4:** Between-group comparison of pretest–post-test change in self-efficacy.

Group	Change Score mdn (IQR)	U	*p*	*r*	d
MCII (*n*_*1*_ = 13)	− 0.30 (− 0.45–0.00)	94.50	0.015	0.52	1.22
MCII + Reminders (*n*_*2*_ = 9)	0.20 (0.00–0.20)				

CI for the primary effect size were *r* = 0.52, 95% CI [0.09, 0.78] and *d* = 1.22, 95% CI [0.18, 2.21]. The wide CI reflect sampling variability associated with the small sample size, and effect size estimates derived from small samples may be positively biased. Accordingly, the observed magnitude should be interpreted cautiously and considered preliminary pending replication in larger samples. A Mann–Whitney *U* test comparing session completion between groups revealed no statistically significant difference in attendance (MCII-only: median = 3.0; MCII + Reminders: median = 3.0; *U* = 51.50, *p* = 0.44, *r* = 0.17). This suggests that the observed self-efficacy differences are unlikely to be explained by differential intervention exposure. Reminder messages did not substantially influence attendance; however, they may have influenced participants’ cognitive engagement with the MCII exercise prior to training sessions.

## Discussion

This study sought to examine whether adding WhatsApp reminder messages to a contextually adapted MCII intervention was associated with changes in self-efficacy among university students in tennis training. The present findings indicate that participants who received MCII combined with reminder messages exhibited more favourable changes in self-efficacy than those who received MCII alone, although neither intervention condition produced a statistically significant within-group change.

These findings suggest that integrating digital reminder messages into MCII-based interventions in sport practice may support improvements in self-efficacy related to goal-directed behaviour. This finding is partially consistent with research showing that the mental contrasting component of MCII can transform positive fantasies about desired futures into binding goals by prompting individuals to consider obstacles that may impede goal attainment^20,21,56^. Likewise, as other researchers have suggested, the implementation intention component of MCII can strengthen goal pursuit by linking anticipated situations to goal-directed responses^29,31,34,57–59^. However, most of the previous studies have investigated the effectiveness of mental contrasting^23–25^ or implementation intentions^39,59,60^ separately, and relatively fewer studies have explored the combined use of these strategies within MCII interventions^59^. To our knowledge, no previous study has examined the role of digitally delivered reminder messages (e.g., WhatsApp) in supporting MCII-based interventions in a sport training context such as tennis. Thus, the findings of this study contribute to the literature by extending MCII research into a real-world sport training context and by examining the potential role of digitally delivered reminder messages in supporting self-regulation processes and self-efficacy development in goal pursuit.

The findings also reveal that the MCII-only condition did not produce a statistically detectable increase in self-efficacy. This result is not fully consistent with the general view in the literature, which suggests that MCII interventions can strengthen goal pursuit and self-regulation^9,36,37^, whilst its effects on self-efficacy have been less frequently examined. This could be explained by the fact that previous studies often examined MCII effects under controlled conditions or focused on goal attainment outcomes, rather than investigating how such interventions function during ongoing sport training activities. Moreover, as Jones^61^, Kettunen et al.^62^ and Wright et al.^63^ have suggested, the effectiveness of behavioural interventions may depend not only on the intervention content but also on the manner and context in which it is delivered (e.g., digitally mediated formats), which may shape individuals’ confidence, self-efficacy, and engagement with goal-directed behaviour. Ascertaining other reasons for these tendencies opens up new directions for future research.

Specifically, the results of the study indicate that the most pronounced effect concerned the difference between interventions rather than a stand-alone improvement within either group. This finding also suggests that whilst this study provides empirical evidence for the comparative advantage of the reminder-supported format, neither intervention produced significant within-group change on its own. The intervention therefore demonstrated a relative advantage rather than a strong standalone effect. The absence of standalone improvement in either condition contrasts with prior MCII controlled studies (e.g., Kirk et al.^35^). This discrepancy may reflect the unpredictable obstacles inherent in real-world sport training, which can dilute the effects of brief interventions. However, implementing the intervention within an ongoing training environment may enhance the ecological validity of the findings by capturing behavioural processes as they occur in a natural sport practice context^64^. In such contexts, reminder messages may help sustain cognitive engagement with goal intentions, which may account for their relative advantage over the MCII-only condition. Consistent with implementation intention theory, reminder messages may function as situational cues that help activate goal-directed responses, thereby facilitating the enactment of goal intentions during practice^29^. In addition, from a self-efficacy perspective, these findings may also be interpreted through Bandura’s social cognitive theory^9,36^. Regular participation in sporting activities may provide opportunities for mastery experiences, which are widely considered the most influential source of efficacy beliefs. Reminder messages, in turn, may function as a form of verbal persuasion that encourages persistence and sustained effort during practice.

Moreover, the results reflect that session attendance did not differ between groups. This finding suggests that greater participation does not explain the self-efficacy change in the reminder-supported condition. Since cognitive engagement is a key mechanism in self-regulation interventions, this finding is consistent with the research showing that MCII effectiveness may depend on individual factors such as goal motives^45,46^, threat appraisals^46^, and obstacles encountered during goal pursuit^45^. Motivational orientations may also influence how individuals engage with reminder-supported MCII interventions^44^. Research grounded in self-determination theory shows that autonomy-supportive motivational environments and coaching behaviours can enhance participants’ engagement and self-regulatory processes in sport contexts^65–67^.

## Study limitations and future research recommendations

This study has several limitations that should be considered when interpreting the findings. First, the relatively small sample size limited the statistical power of the analyses and reduced the ability to detect small or moderate intervention effects; therefore, the absence of statistically significant within-group changes should be interpreted cautiously. In addition, effect size estimates derived from small samples may be unstable, and the wide CI observed in the present study indicate uncertainty regarding the true magnitude of the intervention effect. Second, self-efficacy was assessed using the GSES rather than a tennis-specific instrument. Although the GSES captures broad self-regulatory beliefs, a domain-specific measure might have been more sensitive to changes in sport-related confidence. Third, participant attrition during the intervention period represents another potential limitation. Because the primary analyses relied on complete-case data, participants who discontinued participation may have differed systematically from those who completed the intervention. Although an exploratory intention-to-treat sensitivity analysis produced a similar pattern of results, the small sample size limits firm conclusions regarding potential attrition effects. Fourth, participants were recruited from a single university tennis club using a convenience sampling strategy, which may limit the generalizability of the findings to other sports contexts or athlete populations. Finally, the study compared two intervention formats without including a non-intervention control group; therefore, the results should be interpreted as demonstrating the relative advantage of reminder-supported MCII compared with MCII alone rather than the absolute effectiveness of MCII relative to no intervention.

Future research should aim to replicate these findings using larger samples and more robust experimental designs. In particular, studies including a non-intervention control group and longer intervention periods may help clarify the independent and sustained effects of MCII-based interventions. In addition, future studies may benefit from incorporating sport-specific self-efficacy measures and examining the integration of MCII with digital reminder systems across different sport settings. Since the present study provides one of the first empirical examinations of MCII combined with digital reminder messages in a sport training context, further research is needed to determine whether this approach represents a stable and generalizable strategy for supporting self-regulation in sport practice contexts.

## Conclusion

This study examined whether adding WhatsApp reminder messages to an MCII intervention adapted for a sport training context was associated with changes in university students’ self-efficacy during tennis training. The findings indicate that reminder-supported MCII was associated with more favourable changes in self-efficacy than MCII alone, although neither intervention condition produced statistically significant within-group change. These results provide preliminary evidence that digitally delivered reminder messages may enhance the practical application of MCII in sport training contexts. In applied training settings, simple digital reminder systems may therefore help support self-regulation processes and related self-efficacy beliefs during sport practice.

## Supplementary Information

Below is the link to the electronic supplementary material.

Supplementary Material 1

## Data Availability

The datasets generated during and/or analyzed during the current study are available from the corresponding author on reasonable request.
