# Supplementary material for: Effects of WhatsApp reminder-supported mental contrasting with implementation intentions on university students’ self-efficacy in sport training
Source: Sci Rep. 2026 Mar 27;16:10951. doi: 10.1038/s41598-026-46181-x (PMC13039355; doi:10.1038/s41598-026-46181-x)
Supplement: Supplementary file 1 — Supplementary Material 1 [file 41598_2026_46181_MOESM1_ESM.docx]

Supplementary File S1

Mental Contrasting with Implementation Intentions (MCII) Worksheet for Tennis Training

Participant ID: __________
Date: __________

Please move to a quiet place where you can focus for a few minutes. Take a moment to reflect on your upcoming tennis training session and answer the questions below as clearly and honestly as possible.

# Step 1: Training Goal (Wish)

What is one **specific goal** you want to achieve during this tennis training session?

Your goal should be clear, concrete, and related to your performance or effort during training.

Write your goal here:

|  |
| --- |

# Step 2: Best Outcome

If you successfully achieve this goal during the training session, **what would be the most important positive outcome for you?**

Think about how achieving this goal would benefit your tennis performance or learning.

Write the most important outcome here:

|  |
| --- |

**Visualization instruction**

Take a moment to vividly imagine yourself successfully achieving this goal during the tennis training session. Try to picture the situation as clearly as possible.

# Step 3 — Main Obstacle

Now think about what might prevent you from achieving this goal.

What is the **most important internal or external obstacle** that could interfere with your performance during training?

Examples may include fatigue, loss of concentration, technical difficulty, or lack of motivation.

Write the obstacle here:

|  |
| --- |

**Visualization instruction**

Now imagine the moment when this obstacle might occur during the training session.

# Step 4 — Implementation Intention (If–Then Plan)

How will you respond if this obstacle occurs during training?

Please create a specific **If–Then plan** that links the obstacle with an action you will take.

If ______________________________________________ happens during training,

then I will _____________________________________________.

# Reflection

Before starting the training session, take a moment to mentally review:

- your **training goal**
- the **positive outcome** you want to achieve
- the **obstacle** you identified
- the **If–Then plan** you created

Imagine yourself successfully applying this plan during the tennis training session.
